# Surgery for metastatic spinal differentiated thyroid cancer: feasibility, outcome, and prognostic factors

**DOI:** 10.3389/fsurg.2023.1140150

**Published:** 2023-05-18

**Authors:** Xiajun Liu, Panpan Hu, Shuheng Zhai, Xiao Liu, Ben Wang, Hua Zhou, Xiaoguang Liu, Zhongjun Liu, Feng Wei

**Affiliations:** ^1^ Department of Orthopaedics, Peking University Third Hospital, Beijing, China; ^2^ Engineering Research Center of Bone and Joint Precision Medicine, Ministry of Education, Beijing, China; ^3^ Beijing Key Laboratory of Spinal Disease Research, Beijing, China; ^4^ Department of Spinal Surgery, Baoji Municipal Central Hospital, Baoji, China

**Keywords:** spinal metastases, differentiated thyroid cancer, surgery, survival, prognosis analysis

## Abstract

**Study design:**

A retrospective cohort study.

**Objectives:**

This study aims to report the surgical outcome of metastatic spinal differentiated thyroid cancer (MSDTC) and analyze the factors affecting the prognosis.

**Methods:**

Thirty-five patients were recruited in our single institution who underwent spinal surgery and adjuvant therapies from 2009 to 2019. Two surgical procedures, total *en-bloc* spondylectomy and debulking surgery, were undertaken. Their clinical data, postoperative events, and survival data were collected and analyzed. Survival time and associated factors were further analyzed.

**Results:**

The cohort had a median survival time of 60 months. The mean visual analog scale scores and the Karnofsky performance score improved postoperatively (*p* < 0.05). The patients' Frankel grade was elevated for cases with preoperative neurological deficits (*p* < 0.05). In 31 patients who underwent debulking surgery, 41.9% (*n* = 13) had local recurrences, and radiotherapy reduced the risk of local relapse (*p* < 0.05). Preoperative and postoperative Frankel grades and radioactive iodine (RAI) therapy were associated with the patients’ survival in the univariate analysis (*p* < 0.05). Furthermore, a multivariate regression analysis showed the postoperative Frankel grade as an independent prognostic factor.

**Conclusion:**

Pain, quality of life, and neurological status of patients can be effectively improved after surgery. Radiotherapy can reduce the risk of local recurrences, whereas RAI therapy has a limited effect on local and extraspinal tumor control. Neurological status was independently associated with the patients' survival.

## 1. Introduction

Thyroid cancer is a common endocrine malignancy, with estimated 44,000 new cases per year in the United States (1). Differentiated thyroid cancer (DTC) is the most frequent subtype, and the treatment outcome of primary DTC is usually favorable, with a 5-year survival rate of up to 98.3% (2, 3). However, its poor prognosis is doomed when distant metastasis occurs (4, 5). The bone is one of the most common metastatic sites, occurring in 2%–13% of DTC patients, and half of the bony metastases involve the spinal column (6–8). Patients with metastatic spinal DTC (MSDTC) might develop serious skeletal-relevant events, which generally result in high mortality and severely impaired quality of life (9–12).

For the primary sites of DTC, the standard treatment strategy is surgical resection followed by either radioactive iodine (RAI) or observation (2). However, treatment strategies for MSDTC are controversial. The currently available treatment includes surgery, RAI, radiotherapy, and anti-tumor drugs. Surgery is usually indicated for patients with spinal instability and neurological deficits, and different surgical procedures have been reported, including percutaneous interventions, debulking surgery, and invasive total *en-bloc* spondylectomy (TES) (13). As for RAI, some authors found that the sensitivity of bony metastasis of DTC is decreased. Kushchayeva et al. (11) analyzed 202 patients and found only 57.8% of DTC lesions in the spine were (131)I avid. Similarly, Farooki et al. reported that only half of the bone lesions were RAI-positive (12). Therefore, the use of RAI either as primary or adjuvant therapy in MSDTC lesions requires further investigation. As the development and wide application of advanced delivering devices of radiotherapy (14, 15), some authors adopt the concept of separation surgery followed by stereotactic body radiotherapy (SBRT) for spinal metastases (16). In our center, surgical strategies for MSDTC are made coherently to the principles of circumferential neurological decompression and maximal debulking of tumor load. Following the established framework of our institutional multidisciplinary treatment (MDT) for spinal tumors, adjuvant therapies, including systemic RAI and locoregional radiotherapy, were schemed and implemented after the operation in most cases. The therapeutic outcomes of our patients are favored according to this current clinical study. Therefore, we present a retrospective review of our case series of MSDTC to describe their treatment outcomes and analyze the predisposing factors associated with the prognosis of the disease, including patients' neurological status, performance scores, and visceral involvement. The latter is the main merit of this study, especially the analysis of adjuvant therapies after the operation.

## 2. Materials and methods

### 2.1. Patients inclusion

This is a single-institutional, retrospective cohort study. The inclusion criteria were (1) spinal lesions being surgically treated in our spinal center; (2) diagnosis of MSDTC by pre- or postoperative pathological examination of the metastatic spinal lesions; (3) regular follow-up over two years or till death; (4) full access to all clinical data. The exclusion criteria were (1) MSDTC lesions that had been surgically treated in other centers; (2) other malignancies; (3) no definite pathological diagnosis made. After the eligibility screening, a cohort of 35 patients who underwent surgeries between December 2009 and December 2019 was recruited. The design and conduct of this retrospective study were approved and supervised by our institutional ethics committee board, and informed consent was obtained from all the participants.

### 2.2. Patients' management

**Figure 1:**
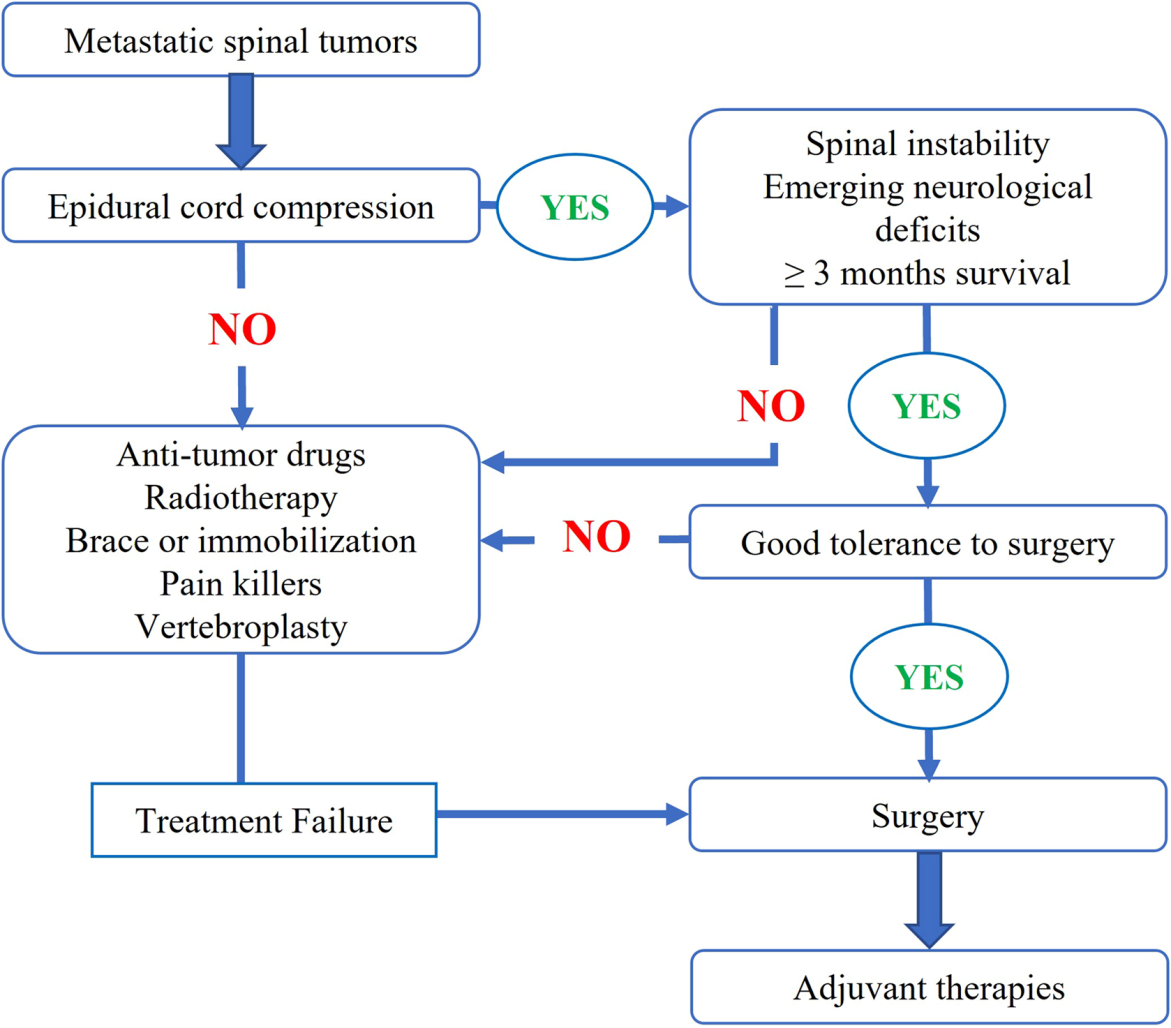
Multidisciplinary treatment algorithm of metastatic spinal tumors in our institution.

Following our institutional multidisciplinary treatment flow of metastatic spinal tumors (Figure 1), surgery was indicated for patients (1) having symptoms and signs of spinal instability, (2) with progressive neurological dysfunction, and/or (3) with severe and refractory local pain. For these patients, preoperative imaging evaluation included x-rays, computed tomography (CT) scans of the spine, and plain and contrast-enhanced magnetic resonance imaging (MRI). In addition, a bone scan or positron emission tomography-computed tomography (PET-CT) was performed to examine the status of any other metastases. For patients with unknown pathologies, we arranged closed (CT-guided in most cases) biopsy procedures to verify the diagnosis.

**Figure 2:**
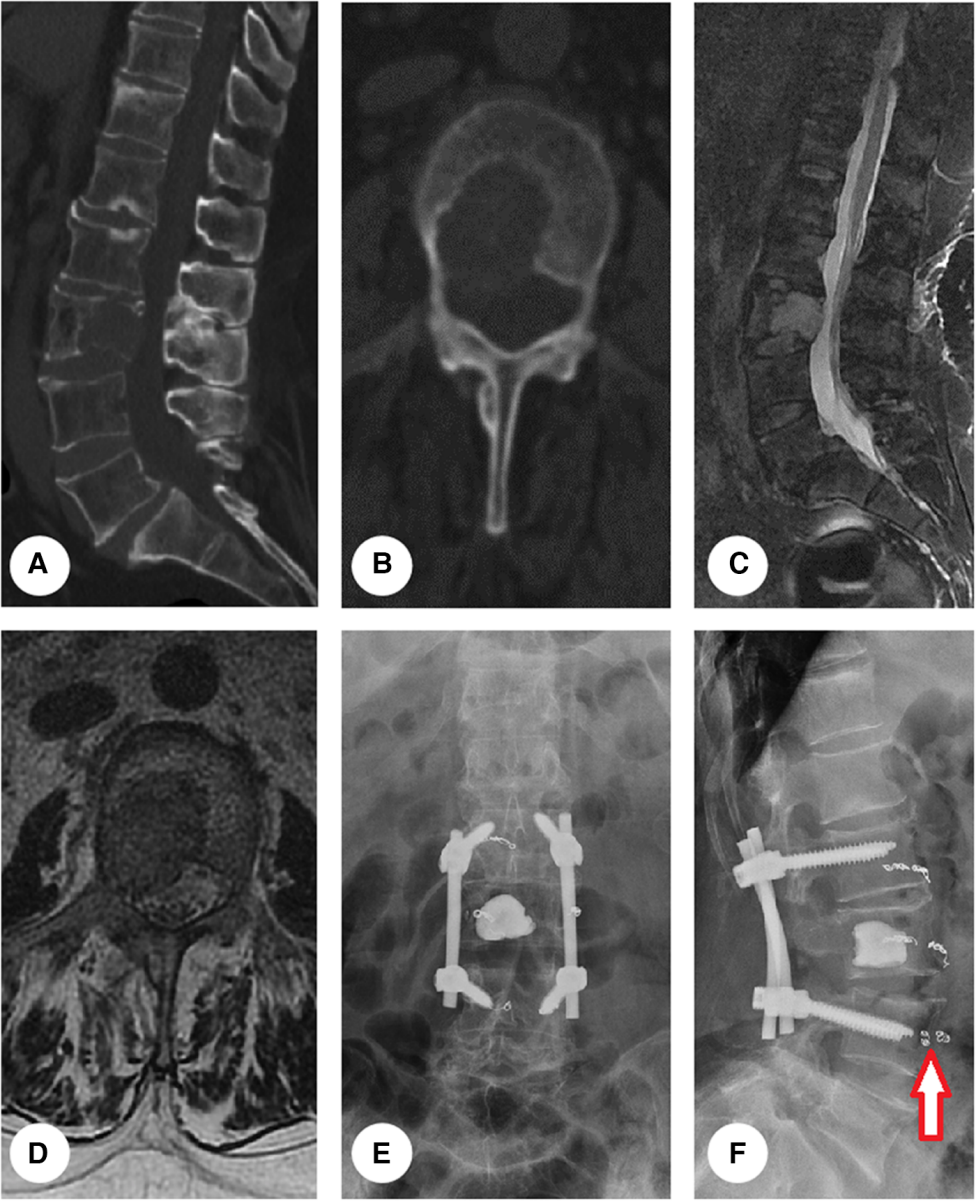
Presentation of representative case. An osteolytic lesion was found within L3 vertebral body (**A,B**). The lesion bulged into the epidural space (**C**) and compressed right L3 nerve root (**D**), arousing severe pain and numbness in right leg. The patient underwent debulking surgery with instrumented fixation (**E,F**). Before the operation, segment feeding vessels were embolized to reduce intraoperative blood loss (red arrow in **F**).

Two surgical procedures were categorized for MSDTC in our cohort: debulking and total excisional surgeries. Total *en-bloc* resection of the metastatic lesions was undertaken in some patients who had solitary spinal lesions and were in good physical condition. For the rest of the cohort, a debulking surgical procedure was performed; during the procedure, we decompressed the cord and the nerve roots, restored the spinal stability via instrumented fixation, and removed the tumor mass as much as possible (Figure 2). After the surgery, the patients were referred to adjuvant therapies, locoregional radiotherapy or systemic RAI accordingly.

### 2.3. Data collection

The clinical records and imaging films of all the patients were carefully reviewed. The main data subsets collected include demographics, surgical procedures, adjuvant therapies, and outcome data. Symptomatically, the pain was assessed by a visual analog scale (VAS), and neurological function was rated according to Frankel scales. Tumor-related details, such as treatment of primary thyroid lesions, history of RAI, systemic metastatic sites (lung, liver, etc.), and time to metastasis/spinal involvement, were also collected. In addition, patients' Karnofsky performance score (KPS), spinal instability neoplastic scores (SINS), and Tomita's and Tokuhashi's prognosis scores were assessed in our practice. Surgical data, such as bleeding volume, operation duration time, and perioperative complications, were also collected.

The primary outcome of this study was patients' survival status. The secondary outcomes included neurological improvement and pain relief, daily performance improvement, local and systemic tumor control, and surgical-related events.

### 2.4. Follow-up strategies

The visit windows were 3, 6, and 12 months after the indexed surgery and then annual life-long assessments. The follow-up time was defined as the interval from the date of spinal surgery to the date of death or the last follow-up. At each visit, patients' neurological function and pain were rated according to VAS and Frankel and KPS systems, and imaging examinations (x-rays, CT, MRI) were performed. PET-CT was recommended when evidence of tumor progression emerged. Follow-up data were collected via outpatient visits and phone interviews.

### 2.5. Statistical analysis

Data analysis was performed using IBM SPSS statistics for Windows Version 20 (IBM Corp., Armonk, NY, USA). Lilliefors test, an adaptation of the Kolmogorov-Smirnov test, was used to examine whether the data were normally distributed. Data were presented as percentages, mean ± standard deviation, or median (range). Two-tailed Mann-Whitney U test and Pearson's *χ*2 test (or Fisher's exact test) were used to compare different groups. We employed Spearman correlation analysis to evaluate the relationship between the interval time to spinal metastasis and patients' survival. The survival rate was plotted according to the Kaplan-Meier method. Potential clinical factors were subjected to univariate and multivariate analyses to identify independent variables to predict prognosis. Univariate analysis was performed via log-rank test, and multivariate analysis was accomplished using a multivariate Cox proportional hazards model, of which the hazard ratio (HR) of each variable and its 95% confidence interval (CI) was displayed. A difference of *p* < 0.05 was considered statistically significant.

## 3. Results

### 3.1. Demographic characteristics

There were 35 cases of MSDTC recruited in this study, and the mean age was 58.7 years (Table 1). Twenty-one (60%) cases had a history of surgery for thyroid tumors, nine of whom had undergone total thyroidectomy, while the other 12 had a subtotal thyroidectomy. Histopathological examinations after the operation were consistent with malignant tumors in 14 patients and benign in the other 7 cases. All the cases of thyroid cancers received RAI as adjuvant therapy for these 14 patients (Table 1). The average interval between surgery of primary thyroid cancers and the finding of MSDTC was 54 months, ranging from 2 to 312 months. Specifically, the interval time to spinal metastasis did not correlate with the patients' survival period (rho = 0.031, *p* = 0.917).

**Table 1:** Demographic data and clinical features of the cohort (*n* = 35).

Items	Values^a^
Age (years)	58.7 ± 10.4
Follow-ups (months)	45.7 ± 12.0
History of thyroidectomy	21 (60.0%)
History of iodine therapy	14 (40.0%)
Preoperative Frankel grades	
B	1 (2.9%)
C	5 (14.3%)
D	8 (22.8%)
E	21 (60.0%)
Preoperative Frankel grades	
C	2 (5.7%)
D	4 (11.4%)
E	29 (82.8%)
Local pain (VAS^#^ scores)	
Preoperative	5.97 ± 1.72
Postoperative	2.43 ± 1.12
Spinal metastatic lesions	
Single vertebra	18 (51.4%)
Multiple vertebrae	17 (48.6%)
Systemic metastasis	
Solitary spinal metastasis	13 (37.1%)
Extraspinal bony metastasis	15 (42.9%)
Concurrent visceral metastasis	11 (31.4%)
Tomita scores	3.7 ± 1.9
Spinal Instability Neoplastic Scores	9.8 ± 2.1
Tokuhashi scores	11.9 ± 2.5
Karnofsky performance scores	
Preoperative	76.0 ± 13.3
Postoperative	89.1 ± 9.5
Surgical procedures	
Total *en-bloc* spondylectomy	4 (11.4%)
Tumor-debulking surgery	31 (88.6%)
Pathologies of spinal lesions	
Follicular	27 (77.1%)
Papillary	8 (22.9%)
Adjuvant therapy after spinal surgery	
Radioactive iodine	25 (71.4%)
Local radiotherapy	17 (48.6%)
Zoledronic acid	8 (22.9%)
None	6 (17.1%)

^a^ Values were presented in the forms mean ± standard deviation or number (percentage), accordingly.

VAS stands for visual analogue scale.

### 3.2. Symptoms and imaging features

The most common symptoms included local pain (34/35, 97.1%) and neurologic deficits (14/35, 40%). The mean VAS score was 5.97 ± 1.72 before the operation (Table 1). The preoperative Frankel grades were E, D, C, and B in 21, 8, 5, and 1 patient, respectively. Six patients lost ambulatory ability before surgery. The mean KPS score before the operation was 76.0 (Table 1).

According to imaging work-up, spinal metastasis involved single vertebral levels in 18 patients and multiple levels in 17 patients. Thirteen cases had solitary spinal lesions without the involvement of extraspinal organs, according to PET-CT scans (Table 1). The cervical spine was involved in 17 cases, 18 in the thoracic spine, 11 in the lumbar spine, and 5 in the sacrum. Thirty patients presented with different degrees of vertebral compression fractures. Based on whole-body screening, 15 patients had other bony metastasis besides spinal lesions, such as the involvement of the ilium, ribs, sternum, and skull. Concurrent visceral metastasis, including the lungs, brain, liver, and pharyngeal lesions, occurred in 31.4% (*n* = 11) of the patients (Table 1). The mean Tomita score was 3.7 ± 1.9 preoperatively. The mean Tokuhashi score was 11.9 ± 2.5. The average SINS score was 9.8 ± 2.1 (Table 1). Closed biopsy was performed preoperatively in 21 patients whose diagnoses were not determined according to medical history and imaging manifestations.

### 3.3. Surgical and adjuvant therapies

The two categories of surgical procedures were performed in our cohort. Four patients with solitary spinal lesions, who were in good physical condition, received TES (Table 1). The other 31 patients received tumor-debulking surgery. Histopathologic examinations revealed the diagnosis of DTC in all cases, with follicular type in 27 (77.1%) patients and papillary type in 8 (22.9%) patients (Table 1).

The mean volume of intraoperative blood loss in the TES group was 1,700 ml, which was significantly higher than that of the debulking group (700 ml, *p* = 0.011). After the operation, five patients (5/31, 16.1%) in the debulking group developed complications, including four cases of muscle weakness and one case of cerebrospinal fluid leakage; three patients (3/4, 75.0%) in the TES group suffered complications of neurological deterioration, pneumothorax, and pleural effusion with atelectasis. The TES group had a higher incidence of postoperative complications than the debulking group (*p* = 0.030). According to the Clavien-Dindo classification, the severity of complications was comparable between the two groups (*p* = 0.523). However, the TES group had a longer postoperative hospital stay than the debulking group, with mean values of 7.5 days and 4.8 days, respectively (*p* < 0.001).

After the surgery, 25 patients (71.4%) received systemic RAI therapy, 17 (48.6%) cases received locoregional radiotherapy, and 8 (22.9%) patients were administrated zoledronic acid (Table 1). Six patients received no adjuvant therapies.

### 3.4. Treatment outcomes

**Figure 3:**
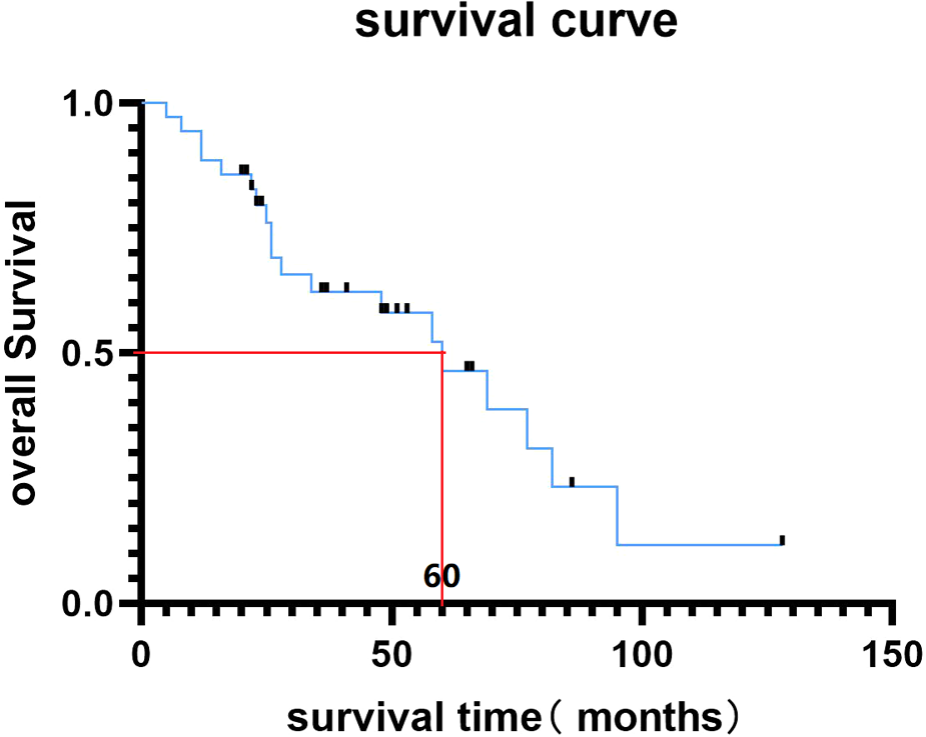
Kaplan-Meier survival curve of MSDTC cases in this study and the median overall survival time was 60 months.

The mean follow-up period was 45.7 months (Table 1). At the last follow-up, 20 (54.3%) patients had died, and 15 patients were still alive. The median overall survival time of our cohort was 60 months (Figure 3). Symptomatically, local pain gained immediate relief in all the patients, and VAS decreased to 2.43 ± 1.12 three months after surgery (Table 1). Five cases developed postoperative neurological deterioration, but they received conservative treatment and recovered to a better neurological status than the preoperative status. During the follow-up, all the patients with neurological deficits experienced the elevation of one or more Frankel grades, and up to 29 (82.8%) patients were rated as Frankel E, namely neurologically intact (Table 1). The postoperative mean KPS score was 89.1 ± 9.5, significantly higher than the preoperative score (*p* < 0.05). Further, the KPS scores were similar between the TES and debulking groups after the operation (*p* = 0.142).

In the TES group, one patient died 95 months after the operation, and the other 3 patients were still alive after 65, 51, and 24 months of follow-up. There was no local recurrence and new viscera metastasis in the TES group, but there was new bony metastasis in the three patients. For the debulking group, the median survival time was 58 months. There were 13 patients with local recurrences, 14 with new bony metastases, and 12 with new viscera metastases (Table 2).

**Table 2:** Outcomes of local and systemic tumor control at the last follow-ups.

	Local recurrences	New bone metastases	New visceral metastases
Surgery (*n* = 35)			
TES^#^ (*n* = 4)	0	3 (75.0%)	0
Debulking surgery (*n* = 31)	13 (41.9%)	14 (45.2%)	12 (38.7%)
*P* value	0.140	0.279	0.169
Radioactive iodine therapy (*n* = 35)			
Yes (*n* = 25)	7 (28.0%)	12 (48.0%)	6 (24.0%)
No (*n* = 10)	6 (60.0%)	5 (50.0%)	6 (60.0%)
*P* value	0.086	0.604	0.053
Local radiotherapy in debulking group (*n* = 31)			
Yes (*n* = 17)	4 (23.5%)	7 (41.2%)	4 (23.5%)
No (*n* = 14)	9 (64.3%)	7 (50.0%)	8 (57.1%)
*P* value	0.022^a^	0.623	0.056

^a^ *P* value less than 0.05 were considered statistically significant.

TES stands for total *en-bloc* spondylectomy.

### 3.5. Follow-up events

Among the 25 patients that underwent RAI therapy were six cases of new viscera metastases, seven cases of local relapse, and 12 cases of new bone lesions during the follow-ups (Table 2). The proportions of local and systemic relapses were higher for the non-RAI group than the RAI group, yet no statistical significance was yielded (Table 2). By comparison, patients who received locoregional radiotherapy had a lower incidence of local recurrence than patients without radiotherapy (*p* = 0.022, Table 2).

### 3.6. Survival-related clinical factors

Factors relating to demographics, neurological status, metastatic sites, viscera involvement, and patients' KPS were introduced into a univariate analysis, among which preoperative Frankel grades (*p* = 0.036), postoperative Frankel grades (*p* < 0.001), and RAI therapy (*p* = 0.037) were significantly associated with survival time (Table 3). Afterward, these survival-associated variables were introduced into a multivariate Cox regression model analysis to confirm independent prognosis-predisposing factors (Table 4). As a result, postoperative Frankel grade was an independent factor affecting patient's survival, with an HR value of 0.184 (95% CI, 0.037–0.908).

**Table 3:** Log-rank test of prognostic factors affecting survival.

Factors	No. of the patients	Survival rate by last follow-up (%)	*P* value
Age (<45y/45–65y/>65y)	4/23/8	100/43.5/25.0	0.055
Sex (male/female)	13/22	53.8/40.9	0.627
Preoperative Frankel grades (A-D/E)	14/21	28.6/57.1	0.036^a^
Histology of DTC^#^ (follicular/papillary)	27/8	48.1/37.5	0.568
Solitary spinal metastases (yes/no)	13/22	53.8/40.9	0.202
Surgical methods (debulking/total spondylectomy)	31/4	41.9/75.0	0.145
Postoperative Frankel grades (A-D/E)	6/29	0/55.2	<0.001^a^
Preoperatively visceral metastases (yes/no)	11/24	36.4/50.0	0.338
Local recurrences (yes/no)	13/22	23.1/59.1	0.406
Postoperatively radioactive iodine therapy (yes/no)	25/10	56.0/20.0	0.037^a^
Postoperatively local radiotherapy (yes/no)	17/18	58.8/33.3	0.422
Postoperatively Zoledronic (yes/no)	8/27	75.0/37.0	0.208

^a^ *P* value less than 0.05 were considered statistically significant.

DTC stands for differentiated thyroid cancer.

**Table 4:** Multivariate cox regression analysis of the prognostic factors affecting survival.

Factors	aHR (95% CI)	*P* Value
Preoperative Frankel grades	0.683 (0.198–2.356)	0.547
Postoperative Frankel grades	0.184 (0.037–0.908)	0.038^a^
Postoperatively radioactive iodine therapy	0.385 (0.121–1.225)	0.106

aHR, adjusted hazard ratio; CI, confidence interval.

^a^ *P* value less than 0.05 were considered statistically significant.

## 4. Discussion

Spinal metastasis of DTC of the follicular type often occurs in patients, although papillary thyroid cancers constitute more than 70% of DTC (4). In our series, 77.1% of patients had follicular DTC, according to the histopathologic examinations (Table 1). DTC is an indolent malignancy; most patients enjoyed a relatively long time of locoregional and systemic tumor control (17). According to our study, the mean interval between the surgery of primary thyroid sites and the diagnosis of MSDTC was 54 months (4.5 years). Therefore, adherence to long and regular systemic monitoring is important for DTC patients; attention should also be paid to spinal metastases during follow-up. Pain and neurological deficits were the most common symptoms in the patients of MSDTC. However, these symptoms are not pathognomonic and hard to differentiate from other metastases (18). For most cases, tumor-related pain presents as the main symptom, and radiotherapy and minimally invasive techniques like spinal cord stimulation (SCS) can provide satisfactory efficacy (19). As most of the lesions were osteolytic, patients with MSDTC had a high risk of pathological fracture, which heavily impaired patients' life quality and neurological function.

Bone metastases have decreased RAI uptake and are less sensitive to radiotherapy. Therefore, the effectiveness of conventional RAI therapy on bony metastasis requires further investigation. Brown et al. (20) reported that DTC with lung metastases has a 10-year survival rate of over 50%, whereas that of bone metastases is 13%–21%. Bernstein et al. (21) reported a clinical trial of 23 patients with MSDTC who received stereotactic radiosurgery; the median survival time was only 28.9 months. We reviewed previous literature on surgery of MSDTC in the databases of Pubmed, Elsevier ScienceDirect, and OVID. We found that the survival time after the operation varied from 15.4 to 50.2 months (Table 5) (22–30). In the current study, the median survival time for our cohort was up to 60 months. Thus, surgical intervention for patients with severe pain and progressive neurological dysfunction is necessary, especially considering the limited efficacy of RAI and external beam radiation therapy (EBRT). According to our study, surgery effectively restores spinal stability and improves patients' Frankel grades (Table 1). The recovery of neurological status after the operation is usually associated with a low risk of life-threatening complications, such as bedsores, urinary and respiratory tract infections, and depression, and higher tolerance to adjuvant therapies, which prolong the patients' life. As this study demonstrated, the postoperative neurological status, rated by Frankel grades, was positively associated with the patients’ survival time (Table 4).

**Table 5:** Comparison of the current study and previously published literature on surgical treatment of spinal metastasis of DTC.

Article	No. of cases	Pathologies	Average follow-up	Surgical strategies	Tumor-related outcomes	Favorable prognostic factors
This study	35	DTC	45.7 months	TES: 4 cases, debulking surgery: 31 cases	Median OS: 60 months	Postoperative Frankel grade
Yin 2021 (18)	50	DTC	2.5 years	Surgery: 16 cases, non-surgery: 34 cases	5-year survival rates: 44.7% for surgical group, 11.1% for non-surgical group	Surgery, local disease control
Liu 2020 (19)	11	DTC	21.8 months	PVP, partial tumor resection	Average OS: 21.8 months	Non-visceral metastasis, open surgery
Zhang 2019 (20)	52	All subtypes	47.1 months	En-bloc resection: 8 cases, curettage: 44 cases	Average OS: 50.2 months	≤50 years old, single segment involved, follicular thyroid cancer
Sellin 2015 (21)	43	All subtypes	39.4 months (median)	Intralesional resection	Median OS: 15.4 months	Preoperative embolization, neurological intact, without progressive systemic disease and complications
Jiang 2014 (22)	21	DTC	42.7 months	TES: 3 cases, palliative curettage: 18 cases	No recurrence for TES, 50% for palliative curettage	No factors verified
Zhang 2013 (23)	22	DTC	30 months	Total resection: 19 cases, partial resection: 3 cases	No evidence of disease: 86.4%	No mentioned
Matsumoto 2013 (24)	8	DTC	6.4 years	TES	No evidence of disease: 62.5%	Not mentioned
Quan 2012 (25)	8	All subtypes	Not calculated	Surgery: 7 cases, non-surgery: 1 case	Mean survival: 39 months	Not mentioned
Demura 2011 (26)	24	All subtypes	55 months	TES: 10 cases, debulking surgery: 14 cases	5-year survival rate: 74%	TES

DTC stands for differentiated thyroid cancer; TES, total en-bloc spondylectomy; PVP, percutaneous vertebroplasty; OS, overall survival.

There were two types of surgical procedures in our case series: TES and palliative debulking surgery. For patients with multi-level spinal lesions or coexistence of extraspinal metastases, debulking surgery combined with neurological decompression and instrumented fixation is indicated. However, the surgical procedure to be undertaken for patients with solitary spinal metastasis is still controversial. Wexler et al. (7) suggested that total spondylectomy is the preferred method in patients with MSDTC who have no apparent visceral metastases and spinal metastases in a single vertebra or two adjoining vertebrae. Demura et al. (30) reviewed a cohort of 24 patients of MSDTC, among whom ten cases underwent TES, and concluded that TES with enough of a margin provided a favorable local control during the patient's lifetime. Among their TES group, there was one case (1/10, 10%) of local recurrence. In our cohort, four patients received TES, and none had local recurrence. However, TES is an invasive procedure and is always complicated with more bleeding, longer operation time, and a higher risk of perioperative complications (31). Furthermore, TES did not decrease the risk of new bony or visceral metastasis. In our series, three of the four TES cases developed new bony metastasis during the follow-ups. Considering that we do not have sufficient evidence to decide whether TES results in longer survival, the pros and cons of TES for patients with solitary spinal metastasis should be carefully considered when making surgical decisions. In recent years, the wide application of 3D prothesis provide reliable spinal reconstruction, which facilitates TES procedure in patients with spinal tumors (32).

RAI therapy is a conventional treatment modality for DTC and systemic metastasis. RAI is advocated as adjuvant therapy to improve long-term outcomes by destroying occult microscopic foci of neoplastic cells within the thyroid remnant or elsewhere in the body (33). However, this practice has also been questioned in the past decade. About two-thirds of patients with distant metastases may show decreased iodine uptake, making adjuvant treatment with RAI ineffective (34). For example, in patients with lung metastases from thyroid carcinoma after RAI therapy, remission rates are quite high (50%–75%), while those of bone metastases are much lower (10%–17%) (6, 20). In our series, compared with the non-RAI group, the patients that received RAI had lower yet insignificant incidences of local relapse and occurence of extraspinal metastasis (Table 2). This study found that RAI marginally decreased the risk of new visceral metastasis in some patients (*p* = 0.053, Table 2). Moreover, RAI therapy was also associated with longer survival time in our univariate analysis (Table 3).

In our case series, 17 patients received EBRT as postoperative adjuvant therapy for better local control (Table 1). We found that the EBRT group had a lower locoregional recurrence rate than the non-EBRT group, though both groups had similar incidences of new extraspinal metastatic lesions (Table 2). Considering the limited effectiveness of RAI therapy on bony metastasis, EBRT remains a good recommendation for patients with debulking surgery. However, considering the proximity of spinal lesions to the cord and other vital structures, the delivery of high-dose SBRT is not easy. Laufer et al. (16) proposed the concept of separation surgery, followed by high-dose hypofractionated stereotactic radiosurgery. This technique emphasized circumferential decompression of the spinal cord and spared a safe zone ventral to the cord for safer delivery of the high radiation dose. In most cases, we adopted the concept of separation surgery. However, though EBRT provided better local tumor control after the operation, this therapy did not prolong the patient's overall survival time (Table 3).

Currently, the prognostic factors which affect the overall survival of MSDTC are unclear. The results of some previous articles in this field were even contradictory (Table 5). Zhang et al. (27) found that age, preoperative and postoperative neurological functions, metastatic sites, histopathologic of DTC, and postoperative RAI therapy were associated with overall survival. Liu et al. (23) found that factors such as viscera metastases and surgical method affected the overall survival time, but age, metastatic sites, histopathologic of DTC, and postoperative radioactive iodine therapy were not associated with overall survival. The current study found that postoperative neurological status was the independent predisposing factor for the patient's survival time (Table 4). For patients with a better neurological status after the operation, they have more chance and will to receive adjuvant systemic RAI or local EBRT therapies in an early postoperative time, which also provides better tumor control.

Our study had some limitations. First, this is a retrospective study which cannot guarantee the homogeneity of the subjects. Second, the sample size is small. This study recruited our single-institutional cases rather than the multicenter cases, so reference to our findings should be done cautiously. Third, some follow-ups were completed via online or smartphone interviews due to the pandemic and travel restrictions; hence, the assessment of neurological status was based on patients' reports. Lastly, this study is a single-arm cohort study; patients who received non-surgical anti-tumor therapies alone were not included as the control group, which affected the level of evidence of this study.

In conclusion, the prognosis of MSDTC is relatively favorable. We found debulking surgery with adjuvant radiotherapy could provide satisfactory local control. Local pain, quality of life, and neurological status of patients were effectively improved after the surgery. Postoperative neurological function is an independent predisposing factor of the prognosis. RAI therapy effectively prevented the occurrence of visceral metastasis but had limited efficacy on metastatic spinal lesions. Thus, a comprehensive therapeutic strategy, composed of surgery, RAI therapy, and radiotherapy, should be considered for the patients with MSDTC.

## Data Availability

The raw data supporting the conclusions of this article will be made available by the authors, without undue reservation.
